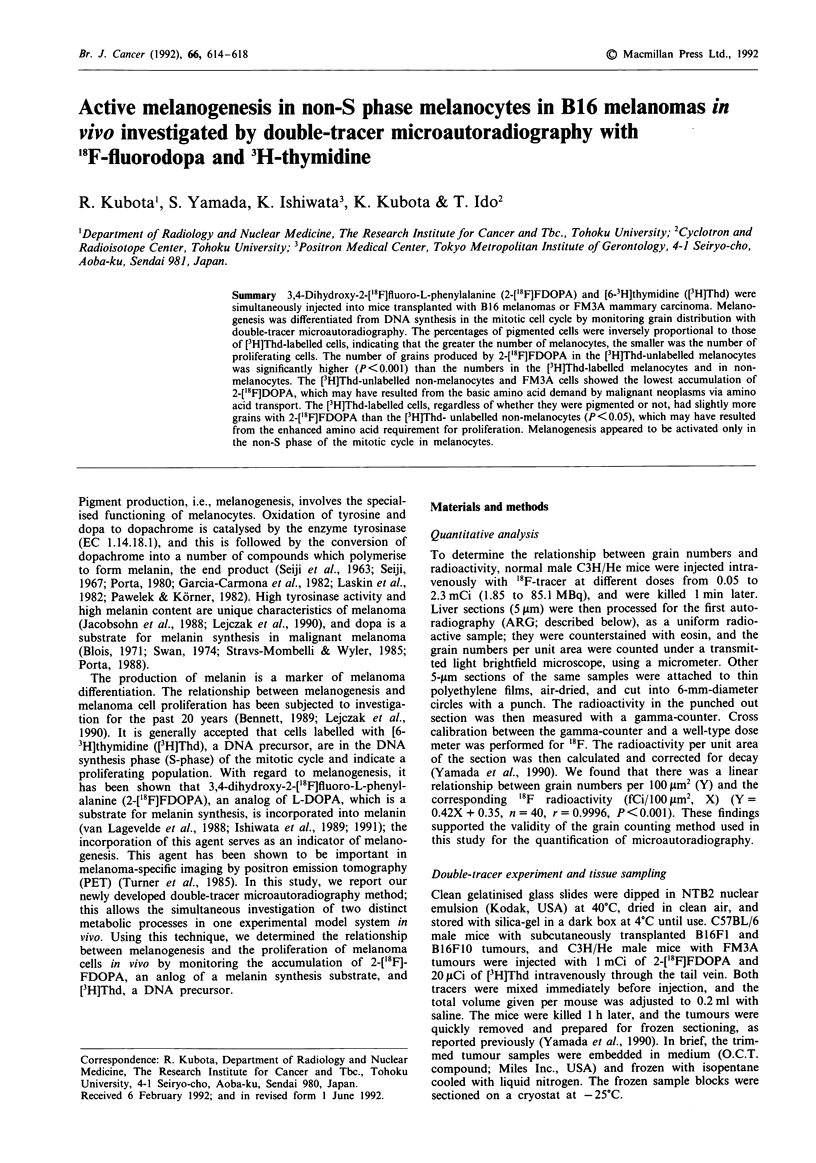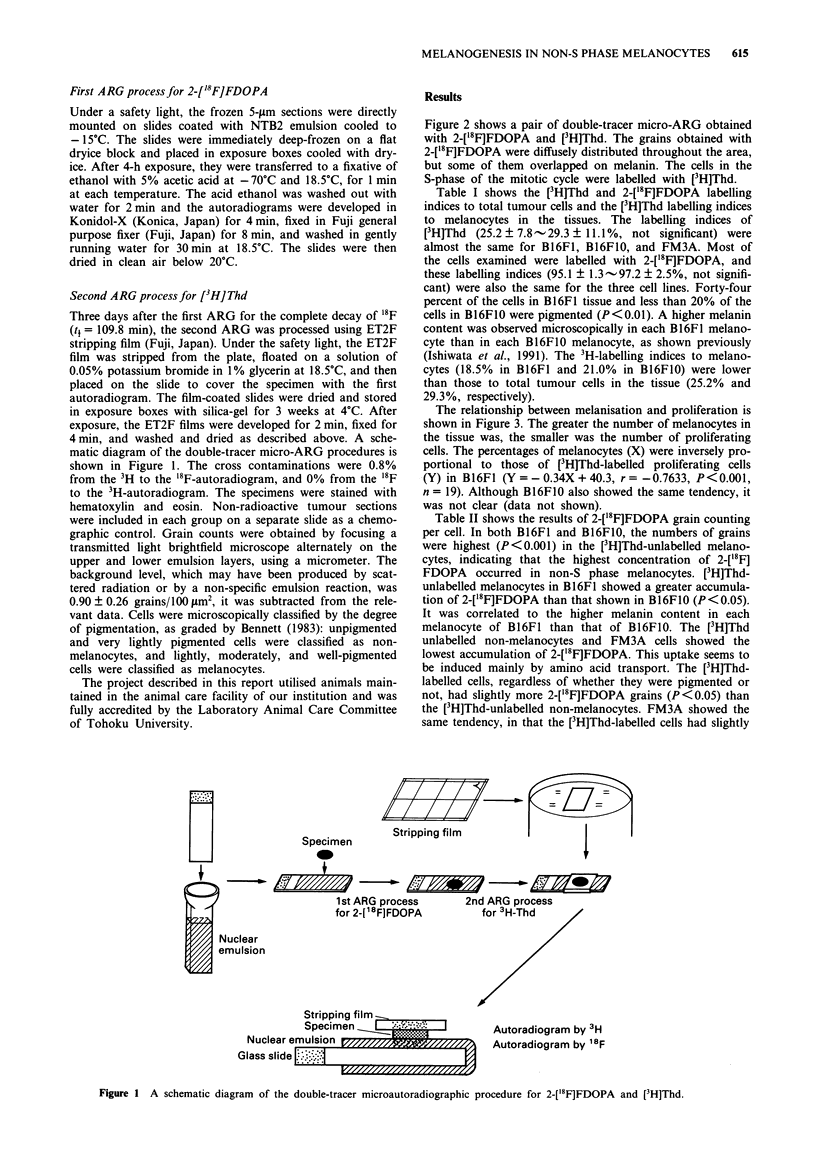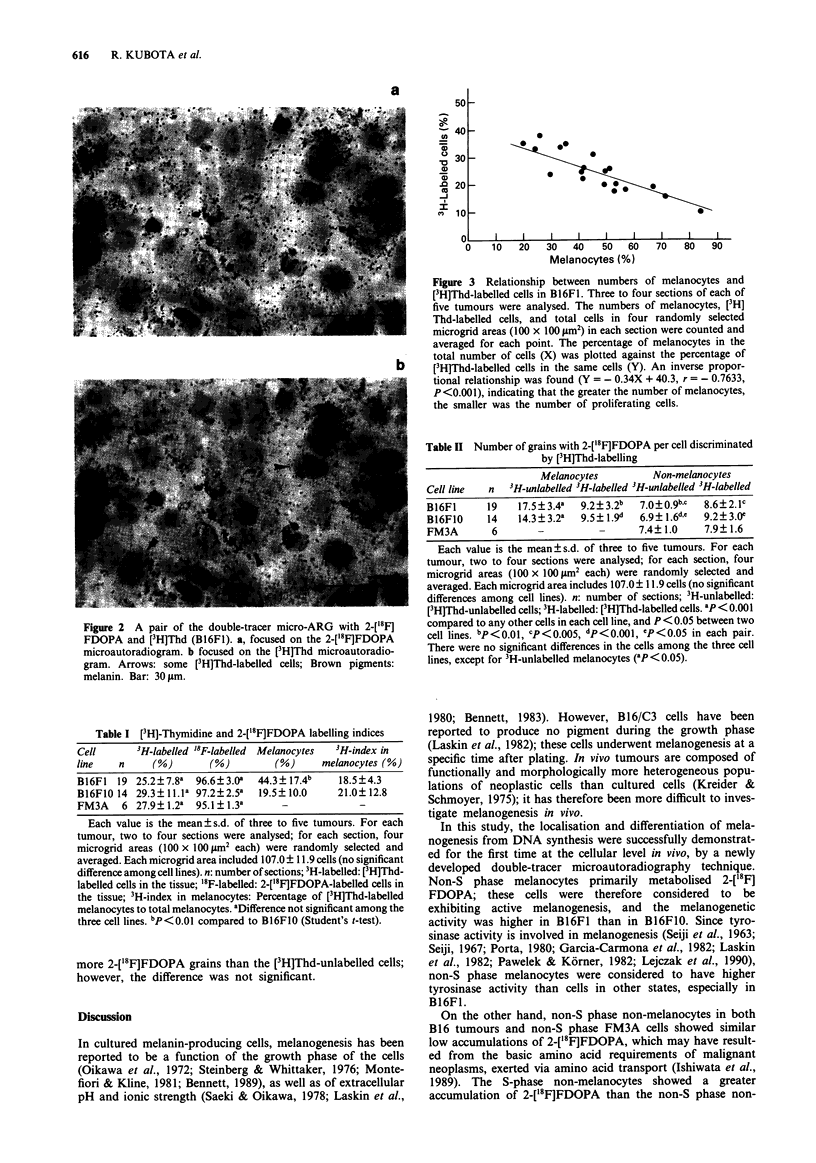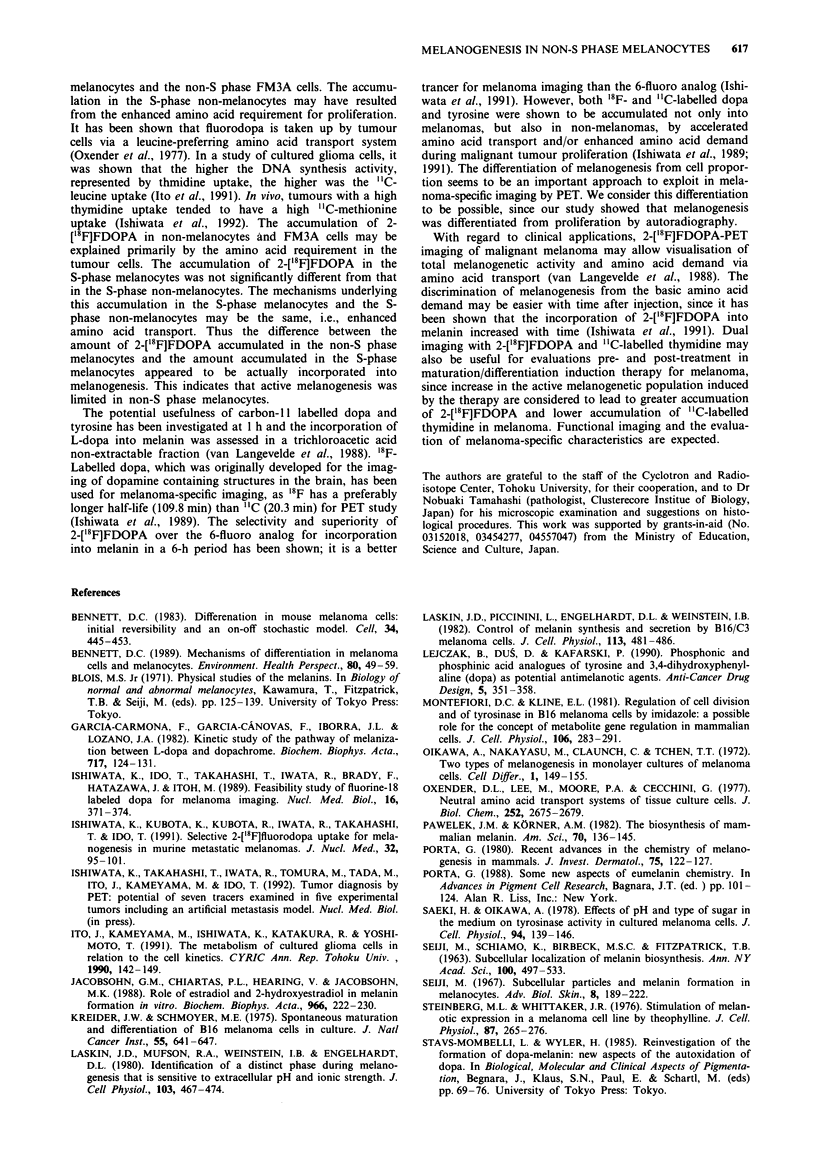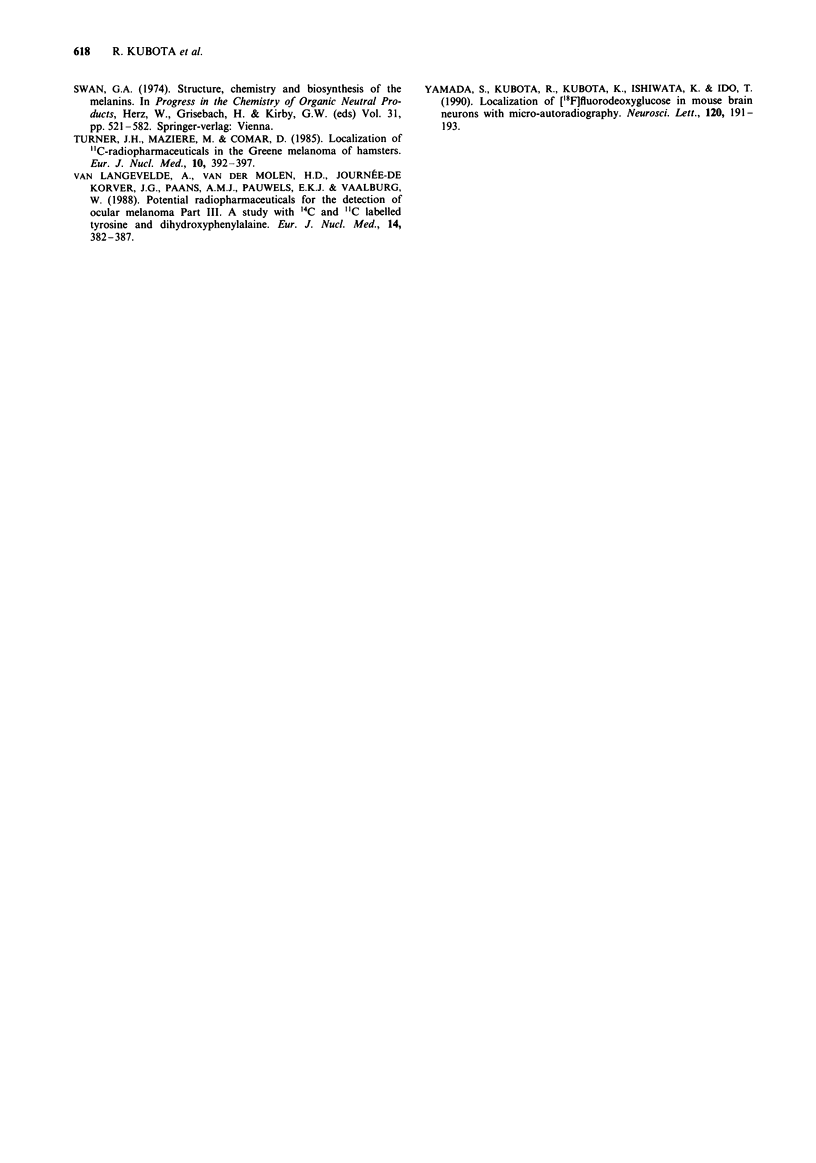# Active melanogenesis in non-S phase melanocytes in B16 melanomas in vivo investigated by double-tracer microautoradiography with 18F-fluorodopa and 3H-thymidine.

**DOI:** 10.1038/bjc.1992.325

**Published:** 1992-10

**Authors:** R. Kubota, S. Yamada, K. Ishiwata, K. Kubota, T. Ido

**Affiliations:** Department of Radiology and Nuclear Medicine, Tohoku University, Sendai, Japan.

## Abstract

**Images:**


*Br. J. Cancer* (1992), **66**, 614–618 © Macmillan Press Ltd., 1992

### Active melanogenesis in non-S phase melanocytes in B16 melanomas *in vivo* investigated by double-tracer microautoradiography with $^{18}$F-fluorodopa and $^{3}$H-thymidine

R. Kubota[1], S. Yamada, K. Ishiwata[3], K. Kubota & T. Ido[2]

[1]*Department of Radiology and Nuclear Medicine, The Research Institute for Cancer and Tbc., Tohoku University;* [2]*Cyclotron and Radioisotope Center, Tohoku University;* [3]*Positron Medical Center, Tokyo Metropolitan Institute of Gerontology, 4-1 Seiryo-cho, Aoba-ku, Sendai 981, Japan.*

**Summary** 3,4-Dihydroxy-2-[$^{18}$F]fluoro-L-phenylalanine (2-[$^{18}$F]FDOPA) and [6-$^{3}$H]thymidine ([$^{3}$H]Thd) were simultaneously injected into mice transplanted with B16 melanomas or FM3A mammary carcinoma. Melanogenesis was differentiated from DNA synthesis in the mitotic cell cycle by monitoring grain distribution with double-tracer microautoradiography. The percentages of pigmented cells were inversely proportional to those of [$^{3}$H]Thd-labelled cells, indicating that the greater the number of melanocytes, the smaller was the number of proliferating cells. The number of grains produced by 2-[$^{18}$F]FDOPA in the [$^{3}$H]Thd-unlabelled melanocytes was significantly higher ($P<0.001$) than the numbers in the [$^{3}$H]Thd-labelled melanocytes and in non-melanocytes. The [$^{3}$H]Thd-unlabelled non-melanocytes and FM3A cells showed the lowest accumulation of 2-[$^{18}$F]DOPA, which may have resulted from the basic amino acid demand by malignant neoplasms via amino acid transport. The [$^{3}$H]Thd-labelled cells, regardless of whether they were pigmented or not, had slightly more grains with 2-[$^{18}$F]FDOPA than the [$^{3}$H]Thd- unlabelled non-melanocytes ($P<0.05$), which may have resulted from the enhanced amino acid requirement for proliferation. Melanogenesis appeared to be activated only in the non-S phase of the mitotic cycle in melanocytes.

Pigment production, i.e., melanogenesis, involves the specialised functioning of melanocytes. Oxidation of tyrosine and dopa to dopachrome is catalysed by the enzyme tyrosinase (EC 1.14.18.1), and this is followed by the conversion of dopachrome into a number of compounds which polymerise to form melanin, the end product (Seiji *et al.*, 1963; Seiji, 1967; Porta, 1980; Garcia-Carmona *et al.*, 1982; Laskin *et al.*, 1982; Pawelek & Körner, 1982). High tyrosinase activity and high melanin content are unique characteristics of melanoma (Jacobsohn *et al.*, 1988; Lejczak *et al.*, 1990), and dopa is a substrate for melanin synthesis in malignant melanoma (Blois, 1971; Swan, 1974; Stravs-Mombelli & Wyler, 1985; Porta, 1988).

The production of melanin is a marker of melanoma differentiation. The relationship between melanogenesis and melanoma cell proliferation has been subjected to investigation for the past 20 years (Bennett, 1989; Lejczak *et al.*, 1990). It is generally accepted that cells labelled with [6-$^{3}$H]thymidine ([$^{3}$H]Thd), a DNA precursor, are in the DNA synthesis phase (S-phase) of the mitotic cycle and indicate a proliferating population. With regard to melanogenesis, it has been shown that 3,4-dihydroxy-2-[$^{18}$F]fluoro-L-phenylalanine (2-[$^{18}$F]FDOPA), an analog of L-DOPA, which is a substrate for melanin synthesis, is incorporated into melanin (van Lagevelde *et al.*, 1988; Ishiwata *et al.*, 1989; 1991); the incorporation of this agent serves as an indicator of melanogenesis. This agent has been shown to be important in melanoma-specific imaging by positron emission tomography (PET) (Turner *et al.*, 1985). In this study, we report our newly developed double-tracer microautoradiography method; this allows the simultaneous investigation of two distinct metabolic processes in one experimental model system *in vivo*. Using this technique, we determined the relationship between melanogenesis and the proliferation of melanoma cells *in vivo* by monitoring the accumulation of 2-[$^{18}$F]-FDOPA, an anlog of a melanin synthesis substrate, and [$^{3}$H]Thd, a DNA precursor.

Correspondence: R. Kubota, Department of Radiology and Nuclear Medicine, The Research Institute for Cancer and Tbc., Tohoku University, 4-1 Seiryo-cho, Aoba-ku, Sendai 980, Japan.
Received 6 February 1992; and in revised form 1 June 1992.

### Materials and methods

### *Quantitative analysis*

To determine the relationship between grain numbers and radioactivity, normal male C3H/He mice were injected intravenously with $^{18}$F-tracer at different doses from 0.05 to 2.3 mCi (1.85 to 85.1 MBq), and were killed 1 min later. Liver sections (5 μm) were then processed for the first autoradiography (ARG; described below), as a uniform radioactive sample; they were counterstained with eosin, and the grain numbers per unit area were counted under a transmitted light brightfield microscope, using a micrometer. Other 5-μm sections of the same samples were attached to thin polyethylene films, air-dried, and cut into 6-mm-diameter circles with a punch. The radioactivity in the punched out section was then measured with a gamma-counter. Cross calibration between the gamma-counter and a well-type dose meter was performed for $^{18}$F. The radioactivity per unit area of the section was then calculated and corrected for decay (Yamada *et al.*, 1990). We found that there was a linear relationship between grain numbers per 100 μm$^2$ (Y) and the corresponding $^{18}$F radioactivity (fCi/100 μm$^2$, X) ($Y = 0.42X + 0.35$, $n = 40$, $r = 0.9996$, $P<0.001$). These findings supported the validity of the grain counting method used in this study for the quantification of microautoradiography.

### *Double-tracer experiment and tissue sampling*

Clean gelatinised glass slides were dipped in NTB2 nuclear emulsion (Kodak, USA) at 40°C, dried in clean air, and stored with silica-gel in a dark box at 4°C until use. C57BL/6 male mice with subcutaneously transplanted B16F1 and B16F10 tumours, and C3H/He male mice with FM3A tumours were injected with 1 mCi of 2-[$^{18}$F]FDOPA and 20 μCi of [$^{3}$H]Thd intravenously through the tail vein. Both tracers were mixed immediately before injection, and the total volume given per mouse was adjusted to 0.2 ml with saline. The mice were killed 1 h later, and the tumours were quickly removed and prepared for frozen sectioning, as reported previously (Yamada *et al.*, 1990). In brief, the trimmed tumour samples were embedded in medium (O.C.T. compound; Miles Inc., USA) and frozen with isopentane cooled with liquid nitrogen. The frozen sample blocks were sectioned on a cryostat at −25°C.

*First ARG process for 2-[$^{18}$F]FDOPA*

Under a safety light, the frozen 5-$\mu$m sections were directly mounted on slides coated with NTB2 emulsion cooled to $-15$°C. The slides were immediately deep-frozen on a flat dryice block and placed in exposure boxes cooled with dry-ice. After 4-h exposure, they were transferred to a fixative of ethanol with 5% acetic acid at $-70$°C and 18.5°C, for 1 min at each temperature. The acid ethanol was washed out with water for 2 min and the autoradiograms were developed in Konidol-X (Konica, Japan) for 4 min, fixed in Fuji general purpose fixer (Fuji, Japan) for 8 min, and washed in gently running water for 30 min at 18.5°C. The slides were then dried in clean air below 20°C.

*Second ARG process for [$^3$H]Thd*

Three days after the first ARG for the complete decay of $^{18}$F ($t_{\frac{1}{2}}$ = 109.8 min), the second ARG was processed using ET2F stripping film (Fuji, Japan). Under the safety light, the ET2F film was stripped from the plate, floated on a solution of 0.05% potassium bromide in 1% glycerin at 18.5°C, and then placed on the slide to cover the specimen with the first autoradiogram. The film-coated slides were dried and stored in exposure boxes with silica-gel for 3 weeks at 4°C. After exposure, the ET2F films were developed for 2 min, fixed for 4 min, and washed and dried as described above. A schematic diagram of the double-tracer micro-ARG procedures is shown in Figure 1. The cross contaminations were 0.8% from the $^3$H to the $^{18}$F-autoradiogram, and 0% from the $^{18}$F to the $^3$H-autoradiogram. The specimens were stained with hematoxylin and eosin. Non-radioactive tumour sections were included in each group on a separate slide as a chemographic control. Grain counts were obtained by focusing a transmitted light brightfield microscope alternately on the upper and lower emulsion layers, using a micrometer. The background level, which may have been produced by scattered radiation or by a non-specific emulsion reaction, was 0.90 ± 0.26 grains/100 $\mu$m$^2$, it was subtracted from the relevant data. Cells were microscopically classified by the degree of pigmentation, as graded by Bennett (1983): unpigmented and very lightly pigmented cells were classified as non-melanocytes, and lightly, moderately, and well-pigmented cells were classified as melanocytes.

The project described in this report utilised animals maintained in the animal care facility of our institution and was fully accredited by the Laboratory Animal Care Committee of Tohoku University.

### Results

Figure 2 shows a pair of double-tracer micro-ARG obtained with 2-[$^{18}$F]FDOPA and [$^3$H]Thd. The grains obtained with 2-[$^{18}$F]FDOPA were diffusely distributed throughout the area, but some of them overlapped on melanin. The cells in the S-phase of the mitotic cycle were labelled with [$^3$H]Thd.

Table I shows the [$^3$H]Thd and 2-[$^{18}$F]FDOPA labelling indices to total tumour cells and the [$^3$H]Thd labelling indices to melanocytes in the tissues. The labelling indices of [$^3$H]Thd (25.2 ± 7.8 ~ 29.3 ± 11.1%, not significant) were almost the same for B16F1, B16F10, and FM3A. Most of the cells examined were labelled with 2-[$^{18}$F]FDOPA, and these labelling indices (95.1 ± 1.3 ~ 97.2 ± 2.5%, not significant) were also the same for the three cell lines. Forty-four percent of the cells in B16F1 tissue and less than 20% of the cells in B16F10 were pigmented ($P < 0.01$). A higher melanin content was observed microscopically in each B16F1 melanocyte than in each B16F10 melanocyte, as shown previously (Ishiwata *et al.*, 1991). The $^3$H-labelling indices to melanocytes (18.5% in B16F1 and 21.0% in B16F10) were lower than those to total tumour cells in the tissue (25.2% and 29.3%, respectively).

The relationship between melanisation and proliferation is shown in Figure 3. The greater the number of melanocytes in the tissue was, the smaller was the number of proliferating cells. The percentages of melanocytes (X) were inversely proportional to those of [$^3$H]Thd-labelled proliferating cells (Y) in B16F1 ($Y = -0.34X + 40.3$, $r = -0.7633$, $P < 0.001$, $n = 19$). Although B16F10 also showed the same tendency, it was not clear (data not shown).

Table II shows the results of 2-[$^{18}$F]FDOPA grain counting per cell. In both B16F1 and B16F10, the numbers of grains were highest ($P < 0.001$) in the [$^3$H]Thd-unlabelled melanocytes, indicating that the highest concentration of 2-[$^{18}$F] FDOPA occurred in non-S phase melanocytes. [$^3$H]Thd-unlabelled melanocytes in B16F1 showed a greater accumulation of 2-[$^{18}$F]FDOPA than that shown in B16F10 ($P < 0.05$). It was correlated to the higher melanin content in each melanocyte of B16F1 than that of B16F10. The [$^3$H]Thd unlabelled non-melanocytes and FM3A cells showed the lowest accumulation of 2-[$^{18}$F]FDOPA. This uptake seems to be induced mainly by amino acid transport. The [$^3$H]Thd-labelled cells, regardless of whether they were pigmented or not, had slightly more 2-[$^{18}$F]FDOPA grains ($P < 0.05$) than the [$^3$H]Thd-unlabelled non-melanocytes. FM3A showed the same tendency, in that the [$^3$H]Thd-labelled cells had slightly

**Figure 1** A schematic diagram of the double-tracer microautoradiographic procedure for 2-[$^{18}$F]FDOPA and [$^3$H]Thd.

**Figure 2** A pair of the double-tracer micro-ARG with 2-[$^{18}$F]FDOPA and [$^3$H]Thd (B16F1). **a**, focused on the 2-[$^{18}$F]FDOPA microautoradiogram. **b** focused on the [$^3$H]Thd microautoradiogram. Arrows: some [$^3$H]Thd-labelled cells; Brown pigments: melanin. Bar: 30 μm.

**Figure 3** Relationship between numbers of melanocytes and [$^3$H]Thd-labelled cells in B16F1. Three to four sections of each of five tumours were analysed. The numbers of melanocytes, [$^3$H]Thd-labelled cells, and total cells in four randomly selected microgrid areas (100 × 100 μm$^2$) in each section were counted and averaged for each point. The percentage of melanocytes in the total number of cells (X) was plotted against the percentage of [$^3$H]Thd-labelled cells in the same cells (Y). An inverse proportional relationship was found (Y = − 0.34X + 40.3, $r = -0.7633$, $P < 0.001$), indicating that the greater the number of melanocytes, the smaller was the number of proliferating cells.

**Table I** [$^3$H]-Thymidine and 2-[$^{18}$F]FDOPA labelling indices

| *Cell line* | *n* | *$^3$H-labelled (%)* | *$^{18}$F-labelled (%)* | *Melanocytes (%)* | *$^3$H-index in melanocytes (%)* |
|---|---|---|---|---|---|
| B16F1 | 19 | 25.2±7.8$^a$ | 96.6±3.0$^a$ | 44.3±17.4$^b$ | 18.5±4.3 |
| B16F10 | 14 | 29.3±11.1$^a$ | 97.2±2.5$^a$ | 19.5±10.0 | 21.0±12.8 |
| FM3A | 6 | 27.9±1.2$^a$ | 95.1±1.3$^a$ | – | – |

Each value is the mean±s.d. of three to five tumours. For each tumour, two to four sections were analysed; for each section, four microgrid areas (100 × 100 μm$^2$ each) were randomly selected and averaged. Each microgrid area included 107.0±11.9 cells (no significant difference among cell lines). *n*: number of sections; $^3$H-labelled: [$^3$H]Thd-labelled cells in the tissue; $^{18}$F-labelled: 2-[$^{18}$F]FDOPA-labelled cells in the tissue; $^3$H-index in melanocytes: Percentage of [$^3$H]Thd-labelled melanocytes to total melanocytes. $^a$Difference not significant among the three cell lines. $^bP < 0.01$ compared to B16F10 (Student's *t*-test).

**Table II** Number of grains with 2-[$^{18}$F]FDOPA per cell discriminated by [$^3$H]Thd-labelling

| *Cell line* | *n* | *Melanocytes* $^3$*H-unlabelled* | *Melanocytes* $^3$*H-labelled* | *Non-melanocytes* $^3$*H-unlabelled* | *Non-melanocytes* $^3$*H-labelled* |
|---|---|---|---|---|---|
| B16F1 | 19 | 17.5±3.4$^a$ | 9.2±3.2$^b$ | 7.0±0.9$^{b,c}$ | 8.6±2.1$^c$ |
| B16F10 | 14 | 14.3±3.2$^a$ | 9.5±1.9$^d$ | 6.9±1.6$^{d,e}$ | 9.2±3.0$^e$ |
| FM3A | 6 | – | – | 7.4±1.0 | 7.9±1.6 |

Each value is the mean±s.d. of three to five tumours. For each tumour, two to four sections were analysed; for each section, four microgrid areas (100 × 100 μm$^2$ each) were randomly selected and averaged. Each microgrid area includes 107.0±11.9 cells (no significant differences among cell lines). *n*: number of sections; $^3$H-unlabelled: [$^3$H]Thd-unlabelled cells; $^3$H-labelled: [$^3$H]Thd-labelled cells. $^aP < 0.001$ compared to any other cells in each cell line, and $P < 0.05$ between two cell lines. $^bP < 0.01$, $^cP < 0.005$, $^dP < 0.001$, $^eP < 0.05$ in each pair. There were no significant differences in the cells among the three cell lines, except for $^3$H-unlabelled melanocytes ($^aP < 0.05$).

more 2-[$^{18}$F]FDOPA grains than the [$^3$H]Thd-unlabelled cells; however, the difference was not significant.

### Discussion

In cultured melanin-producing cells, melanogenesis has been reported to be a function of the growth phase of the cells (Oikawa *et al.*, 1972; Steinberg & Whittaker, 1976; Montefiori & Kline, 1981; Bennett, 1989), as well as of extracellular pH and ionic strength (Saeki & Oikawa, 1978; Laskin *et al.*, 1980; Bennett, 1983). However, B16/C3 cells have been reported to produce no pigment during the growth phase (Laskin *et al.*, 1982); these cells underwent melanogenesis at a specific time after plating. *In vivo* tumours are composed of functionally and morphologically more heterogeneous populations of neoplastic cells than cultured cells (Kreider & Schmoyer, 1975); it has therefore been more difficult to investigate melanogenesis *in vivo*.

In this study, the localisation and differentiation of melanogenesis from DNA synthesis were successfully demonstrated for the first time at the cellular level *in vivo*, by a newly developed double-tracer microautoradiography technique. Non-S phase melanocytes primarily metabolised 2-[$^{18}$F]FDOPA; these cells were therefore considered to be exhibiting active melanogenesis, and the melanogenetic activity was higher in B16F1 than in B16F10. Since tyrosinase activity is involved in melanogenesis (Seiji *et al.*, 1963; Seiji, 1967; Porta, 1980; Garcia-Carmona *et al.*, 1982; Laskin *et al.*, 1982; Pawelek & Körner, 1982; Lejczak *et al.*, 1990), non-S phase melanocytes were considered to have higher tyrosinase activity than cells in other states, especially in B16F1.

On the other hand, non-S phase non-melanocytes in both B16 tumours and non-S phase FM3A cells showed similar low accumulations of 2-[$^{18}$F]FDOPA, which may have resulted from the basic amino acid requirements of malignant neoplasms, exerted via amino acid transport (Ishiwata *et al.*, 1989). The S-phase non-melanocytes showed a greater accumulation of 2-[$^{18}$F]FDOPA than the non-S phase nonmelanocytes and the non-S phase FM3A cells. The accumulation in the S-phase non-melanocytes may have resulted from the enhanced amino acid requirement for proliferation. It has been shown that fluorodopa is taken up by tumour cells via a leucine-preferring amino acid transport system (Oxender *et al.*, 1977). In a study of cultured glioma cells, it was shown that the higher the DNA synthesis activity, represented by thmidine uptake, the higher was the $^{11}$C-leucine uptake (Ito *et al.*, 1991). *In vivo*, tumours with a high thymidine uptake tended to have a high $^{11}$C-methionine uptake (Ishiwata *et al.*, 1992). The accumulation of 2-[$^{18}$F]FDOPA in non-melanocytes and FM3A cells may be explained primarily by the amino acid requirement in the tumour cells. The accumulation of 2-[$^{18}$F]FDOPA in the S-phase melanocytes was not significantly different from that in the S-phase non-melanocytes. The mechanisms underlying this accumulation in the S-phase melanocytes and the S-phase non-melanocytes may be the same, i.e., enhanced amino acid transport. Thus the difference between the amount of 2-[$^{18}$F]FDOPA accumulated in the non-S phase melanocytes and the amount accumulated in the S-phase melanocytes appeared to be actually incorporated into melanogenesis. This indicates that active melanogenesis was limited in non-S phase melanocytes.

The potential usefulness of carbon-11 labelled dopa and tyrosine has been investigated at 1 h and the incorporation of L-dopa into melanin was assessed in a trichloroacetic acid non-extractable fraction (van Langevelde *et al.*, 1988). $^{18}$F-Labelled dopa, which was originally developed for the imaging of dopamine containing structures in the brain, has been used for melanoma-specific imaging, as $^{18}$F has a preferably longer half-life (109.8 min) than $^{11}$C (20.3 min) for PET study (Ishiwata *et al.*, 1989). The selectivity and superiority of 2-[$^{18}$F]FDOPA over the 6-fluoro analog for incorporation into melanin in a 6-h period has been shown; it is a better trancer for melanoma imaging than the 6-fluoro analog (Ishiwata *et al.*, 1991). However, both $^{18}$F- and $^{11}$C-labelled dopa and tyrosine were shown to be accumulated not only into melanomas, but also in non-melanomas, by accelerated amino acid transport and/or enhanced amino acid demand during malignant tumour proliferation (Ishiwata *et al.*, 1989; 1991). The differentiation of melanogenesis from cell proportion seems to be an important approach to exploit in melanoma-specific imaging by PET. We consider this differentiation to be possible, since our study showed that melanogenesis was differentiated from proliferation by autoradiography.

With regard to clinical applications, 2-[$^{18}$F]FDOPA-PET imaging of malignant melanoma may allow visualisation of total melanogenetic activity and amino acid demand via amino acid transport (van Langevelde *et al.*, 1988). The discrimination of melanogenesis from the basic amino acid demand may be easier with time after injection, since it has been shown that the incorporation of 2-[$^{18}$F]FDOPA into melanin increased with time (Ishiwata *et al.*, 1991). Dual imaging with 2-[$^{18}$F]FDOPA and $^{11}$C-labelled thymidine may also be useful for evaluations pre- and post-treatment in maturation/differentiation induction therapy for melanoma, since increase in the active melanogenetic population induced by the therapy are considered to lead to greater accumuation of 2-[$^{18}$F]FDOPA and lower accumulation of $^{11}$C-labelled thymidine in melanoma. Functional imaging and the evaluation of melanoma-specific characteristics are expected.

The authors are grateful to the staff of the Cyclotron and Radioisotope Center, Tohoku University, for their cooperation, and to Dr Nobuaki Tamahashi (pathologist, Clusterecore Institue of Biology, Japan) for his microscopic examination and suggestions on histological procedures. This work was supported by grants-in-aid (No. 03152018, 03454277, 04557047) from the Ministry of Education, Science and Culture, Japan.

### References

BENNETT, D.C. (1983). Differenation in mouse melanoma cells: initial reversibility and an on-off stochastic model. *Cell*, **34**, 445–453.

BENNETT, D.C. (1989). Mechanisms of differentiation in melanoma cells and melanocytes. *Environment. Health Perspect.*, **80**, 49–59.

BLOIS, M.S. Jr (1971). Physical studies of the melanins. In *Biology of normal and abnormal melanocytes*, Kawamura, T., Fitzpatrick, T.B. & Seiji, M. (eds). pp. 125–139. University of Tokyo Press: Tokyo.

GARCIA-CARMONA, F., GARCIA-CÁNOVAS, F., IBORRA, J.L. & LOZANO, J.A. (1982). Kinetic study of the pathway of melanization between L-dopa and dopachrome. *Biochem. Biophys. Acta.*, **717**, 124–131.

ISHIWATA, K., IDO, T., TAKAHASHI, T., IWATA, R., BRADY, F., HATAZAWA, J. & ITOH, M. (1989). Feasibility study of fluorine-18 labeled dopa for melanoma imaging. *Nucl. Med. Biol.*, **16**, 371–374.

ISHIWATA, K., KUBOTA, K., KUBOTA, R., IWATA, R., TAKAHASHI, T. & IDO, T. (1991). Selective 2-[$^{18}$F]fluorodopa uptake for melanogenesis in murine metastatic melanomas. *J. Nucl. Med.*, **32**, 95–101.

ISHIWATA, K., TAKAHASHI, T., IWATA, R., TOMURA, M., TADA, M., ITO, J., KAMEYAMA, M. & IDO, T. (1992). Tumor diagnosis by PET: potential of seven tracers examined in five experimental tumors including an artificial metastasis model. *Nucl. Med. Biol.* (in press).

ITO, J., KAMEYAMA, M., ISHIWATA, K., KATAKURA, R. & YOSHIMOTO, T. (1991). The metabolism of cultured glioma cells in relation to the cell kinetics. *CYRIC Ann. Rep. Tohoku Univ.*, **1990**, 142–149.

JACOBSOHN, G.M., CHIARTAS, P.L., HEARING, V. & JACOBSOHN, M.K. (1988). Role of estradiol and 2-hydroxyestradiol in melanin formation *in vitro*. *Biochem. Biophys. Acta.*, **966**, 222–230.

KREIDER, J.W. & SCHMOYER, M.E. (1975). Spontaneous maturation and differentiation of B16 melanoma cells in culture. *J. Natl Cancer Inst.*, **55**, 641–647.

LASKIN, J.D., MUFSON, R.A., WEINSTEIN, I.B. & ENGELHARDT, D.L. (1980). Identification of a distinct phase during melanogenesis that is sensitive to extracellular pH and ionic strength. *J. Cell Physiol.*, **103**, 467–474.

LASKIN, J.D., PICCININI, L., ENGELHARDT, D.L. & WEINSTEIN, I.B. (1982). Control of melanin synthesis and secretion by B16/C3 melanoma cells. *J. Cell. Physiol.*, **113**, 481–486.

LEJCZAK, B., DUŚ, D. & KAFARSKI, P. (1990). Phosphonic and phosphinic acid analogues of tyrosine and 3,4-dihydroxyphenylaline (dopa) as potential antimelanotic agents. *Anti-Cancer Drug Design*, **5**, 351–358.

MONTEFIORI, D.C. & KLINE, E.L. (1981). Regulation of cell division and of tyrosinase in B16 melanoma cells by imidazole: a possible role for the concept of metabolite gene regulation in mammalian cells. *J. Cell. Physiol.*, **106**, 283–291.

OIKAWA, A., NAKAYASU, M., CLAUNCH, C. & TCHEN, T.T. (1972). Two types of melanogenesis in monolayer cultures of melanoma cells. *Cell Differ.*, **1**, 149–155.

OXENDER, D.L., LEE, M., MOORE, P.A. & CECCHINI, G. (1977). Neutral amino acid transport systems of tissue culture cells. *J. Biol. Chem.*, **252**, 2675–2679.

PAWELEK, J.M. & KÖRNER, A.M. (1982). The biosynthesis of mammalian melanin. *Am. Sci.*, **70**, 136–145.

PORTA, G. (1980). Recent advances in the chemistry of melanogenesis in mammals. *J. Invest. Dermatol.*, **75**, 122–127.

PORTA, G. (1988). Some new aspects of eumelanin chemistry. In *Advances in Pigment Cell Research*, Bagnara, J.T. (ed. ) pp. 101–124. Alan R. Liss, Inc.: New York.

SAEKI, H. & OIKAWA, A. (1978). Effects of pH and type of sugar in the medium on tyrosinase activity in cultured melanoma cells. *J. Cell. Physiol.*, **94**, 139–146.

SEIJI, M., SCHIAMO, K., BIRBECK, M.S.C. & FITZPATRICK, T.B. (1963). Subcellular localization of melanin biosynthesis. *Ann. NY Acad. Sci.*, **100**, 497–533.

SEIJI, M. (1967). Subcellular particles and melanin formation in melanocytes. *Adv. Biol. Skin.*, **8**, 189–222.

STEINBERG, M.L. & WHITTAKER, J.R. (1976). Stimulation of melanotic expression in a melanoma cell line by theophylline. *J. Cell. Physiol.*, **87**, 265–276.

STAVS-MOMBELLI, L. & WYLER, H. (1985). Reinvestigation of the formation of dopa-melanin: new aspects of the autoxidation of dopa. In *Biological, Molecular and Clinical Aspects of Pigmentation*, Begnara, J., Klaus, S.N., Paul, E. & Schartl, M. (eds) pp. 69–76. University of Tokyo Press: Tokyo.

SWAN, G.A. (1974). Structure, chemistry and biosynthesis of the melanins. In *Progress in the Chemistry of Organic Neutral Products*, Herz, W., Grisebach, H. & Kirby, G.W. (eds) Vol. 31, pp. 521–582. Springer-verlag: Vienna.

TURNER, J.H., MAZIERE, M. & COMAR, D. (1985). Localization of $^{11}$C-radiopharmaceuticals in the Greene melanoma of hamsters. *Eur. J. Nucl. Med.*, **10**, 392–397.

VAN LANGEVELDE, A., VAN DER MOLEN, H.D., JOURNÉE-DE KORVER, J.G., PAANS, A.M.J., PAUWELS, E.K.J. & VAALBURG, W. (1988). Potential radiopharmaceuticals for the detection of ocular melanoma Part III. A study with $^{14}$C and $^{11}$C labelled tyrosine and dihydroxyphenylalaine. *Eur. J. Nucl. Med.*, **14**, 382–387.

YAMADA, S., KUBOTA, R., KUBOTA, K., ISHIWATA, K. & IDO, T. (1990). Localization of [$^{18}$F]fluorodeoxyglucose in mouse brain neurons with micro-autoradiography. *Neurosci. Lett.*, **120**, 191–193.